# Conceptions of Happiness Mediate the Relationship Between the Dark Triad and Well-Being

**DOI:** 10.3389/fpsyg.2021.643351

**Published:** 2021-05-11

**Authors:** Mohsen Joshanloo

**Affiliations:** Department of Psychology, Keimyung University, Daegu, South Korea

**Keywords:** dark triad, conceptions of happiness, well-being, machiavellianism, psychopathy, narcissism

## Abstract

This study explored the associations between the Dark Triad traits (i.e., Machiavellianism, psychopathy, and narcissism) and mental well-being in a Korean adult sample (*N* = 1,177). The role of the conceptions of happiness as mediators of these associations was also investigated. Eight conceptions of happiness (e.g., fragility of happiness and eudaimonism), 3 dimensions of hedonic well-being (e.g., life satisfaction), and 2 dimensions of eudaimonic well-being (e.g., psychological well-being) were included in the study. The results showed that Machiavellianism and psychopathy were negatively and narcissism was positively associated with well-being. The pattern of associations between the dark triad and conceptions of happiness showed that Machiavellianism and psychopathy were associated with valuing personal happiness but also considering it to be out of one’s control, and to have negative consequences when achieved. People high on narcissism endorsed generally positive notions of happiness. Results showed that conceptions of happiness functioned as partial mediators in the relationships between the dark triad and well-being. Hence, it seems that the dark triad traits provide a context for the formation of certain beliefs surrounding the nature and value of well-being, which per se influence experienced levels of well-being.

## Introduction

The Dark Triad (DT) refers to the three socially aversive, yet non-pathological, traits of Machiavellianism, psychopathy, and narcissism (Paulhus and Williams, 2002). The DT traits predict a wide range of workplace, educational, mating, interpersonal, and antisocial behaviors and tendencies (Furnham et al., 2013) as well as mental well-being (Aghababaei and Błachnio, 2015). In the present study, it was hypothesized that the lay conceptions of happiness (i.e., beliefs about the nature, value, antecedents, and outcomes of happiness; Joshanloo, 2019) would mediate the relationship between the DT and mental well-being. Eight conceptions of happiness were included in this study. The outcome variables of the study consisted of three hedonic (life satisfaction, positive affect, and negative affect) and two eudaimonic (psychological and social) elements of well-being. All variables of the study are defined in Table 1.

**TABLE 1:** Definitions of the variables used.

Domain	Variable	Definition
Dark triad	Machiavellianism	A selfish orientation in which an individual is willing to use any means to attain goals
	Psychopathy	Characterized by impulsivity, thrill-seeking, callousness, and aggression
	Narcissism	Exaggerated feelings of self-absorption, grandiosity, and entitlement
Conceptions of happiness	Eudaimonism	That well-being consists of meaningful activity, actualizing one’s potential, and understanding the meaning of life rather than hedonic experiences
	Inclusive happiness	That our happiness depends on the happiness and well-being of other people, animals, and the natural elements
	Externality of happiness	That one’s level of happiness is out of one’s control and largely depends on external factors
	Fear of happiness	That happiness can cause bad things to happen
	Transformative suffering	That unhappiness has positive and sometimes transformative powers, and can contribute to well-being
	Fragility of happiness	That happiness is fleeting and may easily turn into less favorable states
	Valuing happiness	That obtaining and maintaining happiness is very important
	Inflexibility of happiness	That one’s level of happiness is fixed and unchangeable
Well-being	Life satisfaction	General satisfaction with present life
	Positive affect	High frequency of positive affect in the past month
	Negative affect	High frequency of negative affect in the past month
	Psychological well-being	Presence of psychological skills and qualities (such as meaning in life and positive relationships)
	Social well-being	Having a positive relationship with one’s communities and society

*Definitions are from Zeigler-Hill and Marcus, 2016; Joshanloo, 2019.*

Machiavellianism and psychopathy have been found to be negatively correlated with various aspects of subjective well-being, eudaimonic well-being, and mental toughness (Egan et al., 2014; Aghababaei and Błachnio, 2015; Wang et al., 2018; Papageorgiou et al., 2019; Aminnuddin, 2020; Limone et al., 2020). Narcissism, however, has shown positive associations with well-being and mental toughness (e.g., Egan et al., 2014; Aghababaei and Błachnio, 2015; Zajenkowski and Czarna, 2015; Giacomin and Jordan, 2016; Papageorgiou et al., 2019; Aminnuddin, 2020). Not every previous study supports the conclusions that Machiavellianism and psychopathy are negatively and narcissism is positively associated with well-being. For example, Wang et al. (2018) found a negative association between narcissism and meaning in life, and Brand et al. (2016) found positive correlations between Machiavellianism and psychopathy and mental toughness.

Conceptions of happiness are also associated with experienced mental well-being (Joshanloo, 2014; Joshanloo et al., 2016). In a study including eight conceptions of happiness, Joshanloo (2019) found that these conceptions collectively explained between 16% and 29% of the variance in various well-being dimensions. The relationship between the conceptions of happiness and the DT has not been investigated in prior research and is the focus of the present study. The DT traits seem to have implications for how people define well-being and success for themselves. For example, Jonason and Tome (2019) showed that the DT traits partly determine projections of happiness in response to imagined life events (e.g., earning status/power or finding a new romantic partner). The DT traits are also associated with endorsing certain values that have implications for conceptions of well-being. For example, Kajonius et al. (2015) showed that Machiavellianism and narcissism were positively associated with the personal values of achievement and power, whereas psychopathy was positively associated with hedonism and power. Thus, people high in the DT may endorse ego-centric, asocial, or antisocial notions of well-being where their personal happiness is prioritized over other people’s happiness.

Although the direction of causality has not been investigated between these variables, and cannot be addressed in cross-sectional studies, given that personality traits are generally more deep-rooted and stable than beliefs, the direction of causality is more likely to run from the DT to the conceptions of happiness. The DT traits are fairly stable over time (Zettler et al., 2020) and there is evidence of their high levels of heritability (Schermer and Jones, 2020). Lau and Marsee (2012), for example, showed that DT-related behaviors were already evident in ages 11–17. Thus, along with other personality traits and environmental influences, the DT traits provide the context for the formation of personal beliefs concerning well-being.

### The Present Study

It is hypothesized in this study that conceptions of happiness are among the mediating pathways through which the DT traits influence well-being. One’s level of the DT traits would partly determine an individual’s beliefs surrounding well-being, which per se influence experienced levels of well-being. A Korean sample was used to test this hypothesis. Due to an overall lack of prior literature on the topic, this study sought to explore these associations with no specific hypotheses made *a priori* with regards to the individual conceptions. However, the available evidence reviewed above does seem to support a number of general expectations:

1- Machiavellianism and psychopathy would be negatively and narcissism would be positively associated with well-being.
2- Machiavellianism and psychopathy would be positively associated with the conceptions of happiness that undermine well-being. Machiavellianism and psychopathy would be inversely associated with the conceptions that positively predict well-being.
3- Narcissism would be positively associated with the conceptions of happiness that boost well-being and would be negatively associated with the conceptions that undermine well-being.
4- Conceptions of happiness would partially mediate the relationship between the DT and well-being.

## Method

### Participants

The sample consisted of 1,177 Korean participants (average age = 40.955, *SD* = 12.097, females = 51.1%). The participants were recruited through a data collection agency, and received monetary compensation. Participants who did not respond correctly to the three attention check questions, were excluded. A part of the data was used in a previous study (Joshanloo, 2019) to answer a different research question (i.e., investigating the construct validity of the conceptions of happiness).

### Measures

#### Dark Triad

The Short Dark Triad Questionnaire (Jones and Paulhus, 2014) is a 27-item measure of Machiavellianism (α = 0.786), psychopathy (α = 0.660), and narcissism (α = 0.737), with nine items per subscale. The items are rated on a 5-point scale ranging from 1 = *disagree strongly* to 5 = *agree strongly*. Item 20 was removed from the Psychopathy subscale because it had a weak and negative correlation (*r* = −0.085) with the total sub-scale, and its removal increased the alpha from 0.660 to 0.714.

#### Well-Being

The *Satisfaction With Life Scale* (Diener et al., 1985) was used to measure life satisfaction (α = 0.920). Each of the five items is rated on a 7-point scale ranging from 1 = *strongly disagree* to 7 = *strongly agree*. The *Negative And Positive Affect Scale* (Mroczek and Kolarz, 1998; Joshanloo, 2017a) was used to measure positive and negative affect. The scale includes six items for negative affect (e.g., nervous, α = 0.877) and six items for positive affect (e.g., cheerful, α = 0.924). Respondents indicate how much of the time (ranging from 1 = *none of the time* to 5 = *all of the time*) during the past 30 days they felt each of the affective states. The *Social* and *Psychological Well-Being* subscales of the Mental Health Continuum-Short Form (Keyes, 2006) were used to measure social (five items, α = 0.813) and psychological (six items, α = 0.900) well-being. The items are responded to on a 6-point scale ranging from 0 = *never* to 5 = *every day.* To calculate a global well-being score for each person, negative affect was subtracted from the sum of the other four variables, after z-transforming the five well-being variables^1^.

#### Conceptions of Happiness

The items of the 5-item *Fear of Happiness Scale* (Joshanloo, 2013; Joshanloo et al., 2014) are rated on a 7-point scale ranging from 1 = *strongly disagree* to 7 = *strongly agree*. The 4-item *Externality of Happiness Scale* (Joshanloo, 2017b), the 4-item *Fragility of Happiness Scale* (Joshanloo et al., 2015), the 5-item *Transformative Suffering Scale* (Joshanloo, 2014), the 4-item *Inflexibility of Happiness Scale* (Joshanloo, 2019), and the 7-item *Valuing Happiness Scale* (Mauss et al., 2011) have a response format identical to that of the Fear of Happiness Scale. In the *Eudaimonism And Hedonism Scale* (Joshanloo, 2019), the participants are asked to distribute 100 points among six components of well-being. Three of the components are hedonic and three of them are eudaimonic. In the present study, only the eudaimonic well-being score is used because including both hedonic and eudaimonic variables in multivariate analyses would be inappropriate given a perfect negative correlation between the two. Eudaimonic scores are calculated by summing or averaging the points given to the eudaimonic items. The *Inclusive Happiness Scale* (Joshanloo, 2019) presents seven pairs of circles that range from two non-overlapping circles to nearly congruent circles. Each of the six items asks about the perceived overlap between one’s happiness and the happiness of a group of people, animals, or plants. Alphas for inflexibility, externality, fear, transformative, fragility, inclusive, and valuing were 0.686, 0.802, 0.823, 0.830, 0.842, 0.893, 0.663, respectively^2^.

Items were recoded when needed, such that higher scores indicate higher levels of the variables. The total score for each variable was calculated by averaging the responses to the items of that variable. All of the scales were translated into Korean by a team of bilinguals, research assistants, and university professors using back-translation, collaborative, and iterative translation procedures (Douglas and Craig, 2007).

## Results

### Preliminary Analyses

SPSS and JASP were used for data analysis. The descriptive statistics for all variables are presented in the Supplementary material (Supplementary Table 1). Table 2 presents the intercorrelations between the DT, conceptions, and global well-being. The intercorrelations including the five well-being variables are presented in the Supplementary material (Supplementary Table 2). Five separate regression analyses were conducted with well-being variables as outcomes and the DT variables as predictors. The results are presented in Table 3. As expected, Machiavellianism and psychopathy predicted well-being negatively, whereas narcissism predicted well-being positively. *R*^2^ values ranged between about 11% (negative affect) and 23% (psychological well-being). Next, eight regression analyses were performed where the DT predicted conceptions of happiness. As shown in Table 4, the DT explained approximately between 1% (transformative) and 9% (valuing) of the variance in the conceptions. A regression analysis was conducted with the conceptions and the DT predicting global well-being (*F*(11, 1165) = 61.485, *p* < 0.001). The results are presented in Table 5. Together, the variables explained about 37% of the variance in global well-being. A follow-up hierarchical regression showed that when the DT variables are entered in the first step, they explained 22% of the variance. When entered in the second step, the conceptions contributed about 15% more. A second hierarchical regression showed that the conceptions explained about 26% of the variance in global well-being and the DT in the second step contributed 11% more. The detailed results of these analyses are presented in the Supplementary material. Thus, each set of variables contribute significantly to global well-being when controlling for the other set of variables. However, the contribution of conceptions is slightly larger than that of the DT.

**TABLE 2:** Intercorrelations between the variables of the study.

	1	2	3	4	5	6	7	8	9	10	11
1. Machiavellianism											
2. Psychopathy	0.535***										
3. Narcissism	0.146***	0.215***									
4. Eudaimonism	−0.095**	−0.071*	0.096**								
5. Inflexibility	0.133***	0.078**	0.009	−0.090**							
6. Externality	0.215***	0.217***	–0.016	−0.090**	0.334***						
7. Fear	0.185***	0.214***	–0.038	0.034	0.183***	0.446***					
8. Transformative	0.058*	0.051	0.095**	0.150***	0.001	0.008	0.331***				
9. Fragility	0.189***	0.134***	−0.070*	–0.006	0.012	0.158***	0.223***	0.286***			
10. Inclusive	−0.123***	−0.078***	0.131***	0.116***	–0.024	−0.089**	–0.003	0.174***	−0.081**		
11. Valuing	0.288***	0.208***	0.117***	–0.051	0.135***	0.277***	0.271***	0.187***	0.186***	0.053	
12. Global well-being	−0.207***	−0.216***	0.340***	0.070*	−0.096**	−0.364***	−0.287***	0.077**	−0.249***	0.269***	−.135***

* *p* < .05. ** *p* < .01. *** *p* < .001.

**TABLE 3:** The dark triad predicting well-being.

Outcome	Unstandardized Coefficient	CI: Lower	CI: Upper	*t*	*p*	Standardized coefficient	Semi-partial correlation	*R*^2^, *F, p*
**Life satisfaction**								0.121, 53.851, 0.000
Machiavellianism	–0.238	–0.390	–0.085	–3.062	0.002	–0.099	–0.084	
Psychopathy	–0.422	–0.579	–0.266	–5.283	0.000	–0.174	–0.145	
Narcissism	0.863	0.708	1.017	10.963	0.000	0.308	0.300	
**Positive affect**								0.122, 54.082, 0.000
Machiavellianism	–0.193	–0.277	–0.109	–4.531	0.000	–0.147	–0.124	
Psychopathy	–0.194	–0.281	–0.108	–4.429	0.000	–0.145	–0.121	
Narcissism	0.455	0.370	0.540	10.525	0.000	0.295	0.288	
**Negative affect**								0.111, 48.744, 0.000
Machiavellianism	0.158	0.071	0.246	3.548	0.000	0.116	0.098	
Psychopathy	0.355	0.265	0.446	7.731	0.000	0.255	0.213	
Narcissism	–0.238	–0.326	–0.149	–5.251	0.000	–0.148	–0.145	
**Psychological**								0.235, 120.061,.000
Machiavellianism	–0.151	–0.259	–0.043	–2.743	0.006	–0.083	–0.070	
Psychopathy	–0.356	–0.467	–0.245	–6.275	0.000	–0.192	–0.160	
Narcissism	1.011	0.902	1.121	18.116	0.000	0.474	0.463	
**Social**								0.167, 78.572, 0.000
Machiavellianism	–0.216	–0.319	–0.113	–4.128	0.000	–0.130	–0.110	
Psychopathy	–0.212	–0.318	–0.106	–3.928	0.000	–0.126	–0.105	
Narcissism	0.762	0.658	0.866	14.335	0.000	0.391	0.382	

*All *df*s were 3 and 1,173.*

**TABLE 4:** The dark triad predicting conceptions of happiness.

Outcome	Unstandardized Coefficient	CI: Lower	CI: Upper	*t*	*p*	Standardized coefficient	Semi-partial correlation	*R*^2^, *F, p*
**Eudaimonism**								0.023, 9.280, 0.000
Machiavellianism	–0.791	–1.408	–0.173	–2.513	0.012	–0.086	–0.073	
Psychopathy	–0.474	–1.110	0.162	–1.461	0.144	–0.051	–0.042	
Narcissism	1.286	0.660	1.913	4.028	0.000	0.119	0.116	
**Inflexibility**								0.018, 7.130, 0.000
Machiavellianism	0.241	0.115	0.367	3.759	0.000	0.129	0.109	
Psychopathy	0.021	–0.108	0.151	0.325	0.745	0.011	0.009	
Narcissism	–0.027	–0.155	0.101	–0.416	0.678	–0.012	–0.012	
**Externality**								0.065, 27.360, 0.000
Machiavellianism	0.263	0.141	0.386	4.222	0.000	0.141	0.119	
Psychopathy	0.297	0.171	0.423	4.624	0.000	0.157	0.131	
Narcissism	–0.154	–0.278	–0.030	–2.440	0.015	–0.071	–0.069	
**Fear**								0.061, 25.273, 0.000
Machiavellianism	0.193	0.069	0.317	3.050	0.002	0.102	0.086	
Psychopathy	0.344	0.216	0.472	5.278	0.000	0.179	0.149	
Narcissism	–0.202	–0.328	–0.076	–3.146	0.002	–0.091	–0.089	
**Transformative**								0.011, 4.390, 0.004
Machiavellianism	0.078	–0.056	0.213	1.140	0.254	0.039	0.033	
Psychopathy	0.023	–0.116	0.161	0.319	0.750	0.011	0.009	
Narcissism	0.203	0.067	0.340	2.923	0.004	0.087	0.085	
**Fragility**								0.048, 19.923, 0.000
Machiavellianism	0.314	0.191	0.437	5.007	0.000	0.169	0.143	
Psychopathy	0.127	0.000	0.254	1.963	0.050	0.067	0.056	
Narcissism	–0.238	–0.362	–0.113	–3.733	0.000	–0.109	–0.106	
**Inclusive**								0.039, 16.072, 0.000
Machiavellianism	–0.235	–0.365	–0.105	–3.547	0.000	–0.120	–0.102	
Psychopathy	–0.096	–0.230	0.038	–1.411	0.159	–0.048	–0.040	
Narcissism	0.365	0.233	0.497	5.433	0.000	0.159	0.155	
**Valuing**								0.091, 39.364, 0.000
Machiavellianism	0.306	0.226	0.387	7.430	0.000	0.245	0.207	
Psychopathy	0.079	–0.004	0.163	1.865	0.062	0.062	0.052	
Narcissism	0.100	0.018	0.182	2.382	0.017	0.068	0.066	

*All *df*s were 3 and 1,173.*

**TABLE 5:** The dark triad and conceptions as predictors of global well-being.

	Unstandardized Coefficient	CI: Lower	CI: Upper	*t*	*p*	Standardized coefficient	Semi-partial correlation
Machiavellianism	–0.408	–0.797	–0.018	–2.052	0.040	–0.059	–0.048
Psychopathy	–1.063	–1.456	–0.670	–5.312	0.000	–0.151	–0.124
Narcissism	2.726	2.332	3.119	13.589	0.000	0.337	0.317
Eudaimonism	–0.024	–0.059	0.011	–1.336	0.182	–0.032	–0.031
Inflexibility	0.097	–0.084	0.277	1.050	0.294	0.026	0.024
Externality	–0.802	–1.008	–0.596	–7.639	0.000	–0.217	–0.178
Fear	–0.501	–0.707	–0.296	–4.782	0.000	–0.137	–0.111
Transformative	0.451	0.268	0.633	4.853	0.000	0.130	0.113
Fragility	–0.547	–0.733	–0.362	–5.798	0.000	–0.147	–0.135
Inclusive	0.558	0.389	0.728	6.473	0.000	0.158	0.151
Valuing	–0.218	–0.497	0.062	–1.524	0.128	–0.039	–0.036

### Mediation Analyses

Three mediation analyses were performed using the *Process* macro (Hayes, 2017) with 5,000 bootstrap resamples. In each analysis, the eight conceptions were specified as parallel mediators between one of the DT variables and global well-being, and age and gender were controlled for. Table 6 presents the mediation analysis results. All total, direct, and indirect effects were significant, indicating that partial mediation is supported for Machiavellianism, psychopathy, and narcissism. Significant mediators for Machiavellianism were externality, fear, transformative, fragility, and inclusive. Machiavellianism contributed positively to the first four variables, and negatively to inclusive. For psychopathy, significant mediators were externality, fear, transformative, and fragility. Psychopathy contributed positively to all these variables. Transformative, fragility, inclusive, valuing were significant mediators for narcissism. Narcissism contributed positively to transformative, inclusive, and valuing, and negatively to fragility. Externality, fear, fragility, and valuing contributed negatively, and transformative and inclusive contributed positively to global well-being. Based on these results, it can be concluded that conceptions of happiness mediate the relationship between the DT and global well-being. Except for eudaimonism and inflexibility, all other conceptions were significant mediators for at least one DT trait.

**TABLE 6:** Mediation analyses.

Predictor		Parameter	Unstandardized Effect	SE	95% CI lower	95% CI upper	Mediation supported	P → M	M → O
**Machiavellianism**									
*t* = −6.943	*p* = 0.000	Total	–1.411	0.203	–1.810	–1.012			
*t* = −2.800	*p* = 0.005	Direct	–0.541	0.193	–0.919	–0.162			
		Total Indirect	–0.870	0.137	–1.145	–0.612	✓		
		Eudaimonism	0.000	0.015	–0.028	0.034		−	ns
		Inflexibility	0.031	0.030	–0.027	0.093		+	ns
		Externality	–0.342	0.071	–0.488	–0.214	✓	+	−
		Fear	–0.252	0.061	–0.381	–0.142	✓	+	−
		Transformative	0.101	0.043	0.022	0.194	✓	+	+
		Fragility	–0.244	0.060	–0.373	–0.137	✓	+	−
		Inclusive	–0.133	0.052	–0.243	–0.040	✓	−	+
		Valuing	–0.031	0.067	–0.156	0.104		+	ns
**Psychopathy**									
*t* = −7.325	*p* = 0.000	Total	–1.566	0.214	–1.985	–1.146			
*t* = −3.855	*p* = 0.000	Direct	–0.763	0.198	–1.151	–0.375			
		Total Indirect	–0.803	0.133	–1.076	–0.549	✓		
		Eudaimonism	0.000	0.009	–0.020	0.019		ns	ns
		Inflexibility	0.019	0.022	–0.023	0.065		+	ns
		Externality	–0.365	0.075	–0.529	–0.233	✓	+	−
		Fear	–0.303	0.069	–0.452	–0.181	✓	+	−
		Transformative	0.114	0.046	0.032	0.213	✓	+	+
		Fragility	–0.182	0.057	–0.306	–0.078	✓	+	−
		Inclusive	–0.055	0.050	–0.159	0.040		ns	+
		Valuing	–0.029	0.053	–0.131	0.077		+	ns
**Narcissism**									
*t* = 12.444	*p* = .000	Total	2.766	0.222	2.330	3.202			
*t* = 11.887	*p* = .000	Direct	2.372	0.200	1.981	2.764			
		Total Indirect	0.394	0.133	0.134	0.662	✓		
		Eudaimonism	–0.019	0.022	–0.066	0.024		+	ns
		Inflexibility	0.001	0.008	–0.017	0.019		ns	ns
		Externality	0.024	0.058	–0.093	0.138		ns	−
		Fear	0.059	0.047	–0.028	0.158		ns	−
		Transformative	0.105	0.041	0.032	0.193	✓	+	+
		Fragility	0.092	0.046	0.012	0.191	✓	−	−
		Inclusive	0.195	0.052	0.103	0.305	✓	+	+
		Valuing	–0.065	0.033	–0.139	–0.010	✓	+	−

*P = predictor. M = mediator. O = outcome.*

## Discussion

In line with the prior research (e.g., Aghababaei and Błachnio, 2015), the present results showed that Machiavellianism and psychopathy were negatively and narcissism was positively associated with well-being. The DT traits were better predictors of psycho-social well-being than subjective well-being, which is consistent with evidence that eudaimonic well-being depends more heavily than hedonic well-being on self- and impulse-control capacities (Joshanloo et al., 2021). People who score high on the DT (particularly psychopathy) are likely to show hampered self-control, which is likely to undermine eudaimonic more than hedonic well-being.

Narcissism has been found to predict an array of adaptive outcomes in previous research, which may explain its positive linkage to well-being. For example, narcissism is associated with lower neuroticism (Lahey, 2009), higher assertiveness (Samuel and Widiger, 2008), self-esteem (Sedikides et al., 2004), higher likelihood of being chosen for leadership positions (Dowgwillo et al., 2016), and better dating outcomes (Jonason et al., 2010). However, the positive link between narcissism and well-being can also be looked at from two other angles. Firstly, the scale of narcissism used in this study predominantly measures the relatively adaptive features of narcissism (e.g., grandiosity and positive self-regard), leaving out more maladaptive features (e.g., exploitativeness and narcissistic fragility), which may inflate the associations with well-being. Secondly, narcissists show a tendency for socially desirable responding and self-enhancement biases (John and Robins, 1994; Kowalski et al., 2018), which may affect their responses to well-being questions. It remains for future research to shed further light on the nature of narcissism-well-being linkages.

The present results showed that Machiavellianism and psychopathy were associated with conceptions that undermine well-being, whereas narcissism was generally associated with conceptions that facilitate well-being. The strongest associations were found between the DT and three conceptions valuing, externality, and fear. Thus, Machiavellianism and psychopathy are linked to the cluster of beliefs that indicate a “doubtful pursuit” of happiness (Joshanloo, 2019), where happiness is valued, but is considered to be out of one’s control, and to have negative consequences when achieved. A previous study also showed that callous/unemotional tendencies were associated with conceiving happiness as inflexible, uncontrollable, and external (Tullett and Plaks, 2016).

Therefore, part of the influence of the DT on well-being can be explained by their shared variance with the conceptions of well-being. The mediation analysis results (Table 6) provided broad support for the prediction that conceptions of happiness mediate the relationship between the DT and well-being. It is noteworthy that although the results of the mediation analyses (Table 6) indicate that transformative suffering is a positive pathway to well-being from all DT traits, in the regression analysis reported above (Table 3), only narcissism had a significant relationship with transformative suffering. This is because, in each mediation analysis, only one of the DT traits was included to predict transformative suffering whereas, in the regression analysis, all three traits were included as predictors (i.e., each effect is adjusted for the other two effects). Therefore, the mediating pathway through transformative suffering for Machiavellianism and psychopathy is a weak one and does not hold if the other two traits are controlled for.

In sum, the present results indicate that people high on Machiavellianism and psychopathy do value and pursue happiness, but they have doubts about the consequences of happiness and their levels of personal control over it. It seems that these two traits prompt the individual to take a competitive approach to obtaining happiness rather than a collective and collaborative approach, where even achieving happiness may cause troubles (e.g., happiness may induce a sense of rivalry or envy in others). This cluster of beliefs is compatible with the selfish, nihilistic, and cynical aspects of the DT, accompanied by the perception that the world is a hostile and competitive place (Rauthmann and Kolar, 2013). People high on narcissism, on the other hand, endorsed more positive notions of well-being, which can partly explain narcissism’s positive links with well-being.

The limitations of the study should be acknowledged. For example, given the cross-sectional nature of the study, the direction of causality between the variables could not be ascertained, and thus future longitudinal and experimental studies are certainly needed. Furthermore, in light of the cross-cultural differences reported in the levels of the DT (Jonason et al., 2020), the present results need to be replicated in other national samples before more definitive conclusions can be drawn. Despite these limitations, this study is the first to show that the DT traits are associated with beliefs surrounding well-being, and provide evidence in support of the hypothesis that people with various levels of the DT traits gravitate towards certain notions of well-being that are per se beneficial or detrimental to well-being.

## Data Availability Statement

The raw data supporting the conclusions of this article will be made available by the authors, upon reasonable request.

## Ethics Statement

The studies involving human participants were reviewed and approved by The Institutional Review Board of Keimyung University. The patients/participants provided their written informed consent to participate in this study.

## Author Contributions

The author confirms being the sole contributor of this work and has approved it for publication.

## Conflict of Interest

The author declares that the research was conducted in the absence of any commercial or financial relationships that could be construed as a potential conflict of interest.

## Footnotes

1 Considering that the five well-being variables used in this study are correlated (e.g., Gallagher et al., 2009; also see the Supplementary material), the well-being measures are not multi-faceted, and this study is interested in measuring well-being comprehensively and generally, a composite index of well-being was constructed and used. The five variables had a good internal consistency (α = .84). An exploratory factor analysis suggested that a single-factor model is consistent with the data, with factor loadings ranging between.537 and.811 and variance explained = 54.040%)

2 With the exception of the Valuing Happiness Scale, all of the conceptions of happiness scales used in this study and their scoring information are publicly available at https://bit.ly/3siBWcV
