# Supplementary material for: Conceptions of Happiness Mediate the Relationship Between the Dark Triad and Well-Being
Source: Front Psychol. 2021 May 11;12:643351. doi: 10.3389/fpsyg.2021.643351 (PMC8144451; doi:10.3389/fpsyg.2021.643351)
Supplement: Supplementary file 1 [file Table_1.DOCX]

| Table S1  *Descriptive Statistics* | | | | | | |
| --- | --- | --- | --- | --- | --- | --- |
|  | Minimum | Maximum | Mean | SD | Skewness | Kurtosis |
| Machiavellianism | 1.000 | 5.000 | 3.036 | .571 | -.119 | .687 |
| Psychopathy | 1.000 | 4.375 | 2.252 | .561 | .348 | .306 |
| Narcissism | 1.222 | 4.333 | 2.664 | .487 | -.002 | .200 |
| Eudaimonism | .000 | 33.333 | 14.913 | 5.255 | .030 | .360 |
| Inflexibility | 1.000 | 7.000 | 3.794 | 1.068 | -.242 | -.314 |
| Externality | 1.000 | 7.000 | 3.039 | 1.064 | .333 | .024 |
| Fear | 1.000 | 7.000 | 2.765 | 1.077 | .423 | -.088 |
| Transformative | 1.000 | 7.000 | 3.978 | 1.139 | -.248 | -.191 |
| Fragility | 1.000 | 7.000 | 4.970 | 1.061 | -.689 | 1.150 |
| Inclusive | 1.000 | 6.667 | 2.950 | 1.116 | .536 | -.107 |
| Valuing | 1.857 | 7.000 | 4.681 | .714 | -.103 | .306 |
| Life satisfaction | 1.000 | 7.000 | 3.614 | 1.366 | -.104 | -.807 |
| Positive affect | 1.000 | 5.000 | 2.888 | .750 | -.228 | -.145 |
| Negative affect | 1.000 | 4.833 | 2.102 | .781 | .809 | .388 |
| Social well-being | .000 | 4.800 | 1.630 | .948 | .419 | -.249 |
| Psychological wb | .000 | 5.000 | 2.151 | 1.038 | .140 | -.508 |
| Global well-being | -11.577 | 11.436 | .000 | 3.942 | -.108 | -.229 |

| Table S2  *Pearson's Correlations Between all Variables of the Study* | | | | | | | | | | | | | | | | | | | | | | | | | | | | | | | | |  |
| --- | --- | --- | --- | --- | --- | --- | --- | --- | --- | --- | --- | --- | --- | --- | --- | --- | --- | --- | --- | --- | --- | --- | --- | --- | --- | --- | --- | --- | --- | --- | --- | --- | --- |
| **Variable** |  | **1** | | **2** | | **3** | | **4** | | **5** | | **6** | | **7** | | **8** | | **9** | | **10** | | **11** | | **12** | | **13** | | **14** | | **15** | | **16** |  |
| 1. Machiavellianism | | — |  |  |  |  |  |  |  |  |  |  |  |  |  |  |  |  |  |  |  |  |  |  |  |  |  |  |  |  |  |  |  |
| 1. Psychopathy | | 0.535 | *** | — |  |  |  |  |  |  |  |  |  |  |  |  |  |  |  |  |  |  |  |  |  |  |  |  |  |  |  |  |  |
| 1. Narcissism | | 0.146 | *** | 0.215 | *** | — |  |  |  |  |  |  |  |  |  |  |  |  |  |  |  |  |  |  |  |  |  |  |  |  |  |  |  |
| 1. Eudaimonism | | -0.095 | ** | -0.071 | * | 0.096 | ** | — |  |  |  |  |  |  |  |  |  |  |  |  |  |  |  |  |  |  |  |  |  |  |  |  |  |
| 1. Inflexibility | | 0.133 | *** | 0.078 | ** | 0.009 |  | -0.090 | ** | — |  |  |  |  |  |  |  |  |  |  |  |  |  |  |  |  |  |  |  |  |  |  |  |
| 1. Externality | | 0.215 | *** | 0.217 | *** | -0.016 |  | -0.090 | ** | 0.334 | *** | — |  |  |  |  |  |  |  |  |  |  |  |  |  |  |  |  |  |  |  |  |  |
| 1. Fear | | 0.185 | *** | 0.214 | *** | -0.038 |  | 0.034 |  | 0.183 | *** | 0.446 | *** | — |  |  |  |  |  |  |  |  |  |  |  |  |  |  |  |  |  |  |  |
| 1. Transformative | | 0.058 | * | 0.051 |  | 0.095 | ** | 0.150 | *** | 8.781e -4 |  | 0.008 |  | 0.331 | *** | — |  |  |  |  |  |  |  |  |  |  |  |  |  |  |  |  |  |
| 1. Fragility | | 0.189 | *** | 0.134 | *** | -0.070 | * | -0.006 |  | 0.012 |  | 0.158 | *** | 0.223 | *** | 0.286 | *** | — |  |  |  |  |  |  |  |  |  |  |  |  |  |  |  |
| 1. Inclusive | | -0.123 | *** | -0.078 | ** | 0.131 | *** | 0.116 | *** | -0.024 |  | -0.089 | ** | -0.003 |  | 0.174 | *** | -0.081 | ** | — |  |  |  |  |  |  |  |  |  |  |  |  |  |
| 1. Valuing | | 0.288 | *** | 0.208 | *** | 0.117 | *** | -0.051 |  | 0.135 | *** | 0.277 | *** | 0.271 | *** | 0.187 | *** | 0.186 | *** | 0.053 |  | — |  |  |  |  |  |  |  |  |  |  |  |
| 1. Life satisfaction | | -0.147 | *** | -0.160 | *** | 0.256 | *** | 0.017 |  | -0.057 |  | -0.293 | *** | -0.214 | *** | 0.080 | ** | -0.220 | *** | 0.218 | *** | -0.127 | *** | — |  |  |  |  |  |  |  |  |  |
| 1. Positive affect | | -0.181 | *** | -0.160 | *** | 0.242 | *** | -0.004 |  | -0.078 | ** | -0.300 | *** | -0.246 | *** | 0.085 | ** | -0.193 | *** | 0.231 | *** | -0.105 | *** | 0.670 | *** | — |  |  |  |  |  |  |  |
| 1. Negative affect | | 0.231 | *** | 0.285 | *** | -0.076 | ** | -0.014 |  | 0.071 | * | 0.366 | *** | 0.324 | *** | 0.074 | * | 0.225 | *** | -0.072 | * | 0.307 | *** | -0.468 | *** | -0.535 | *** | — |  |  |  |  |  |
| 1. Social well-being | | -0.140 | *** | -0.111 | *** | 0.345 | *** | 0.121 | *** | -0.083 | ** | -0.211 | *** | -0.135 | *** | 0.103 | *** | -0.170 | *** | 0.294 | *** | -0.002 |  | 0.523 | *** | 0.504 | *** | -0.278 | *** | — |  |  |  |
| 1. Psychological wb | | -0.116 | *** | -0.135 | *** | 0.421 | *** | 0.125 | *** | -0.088 | ** | -0.265 | *** | -0.211 | *** | 0.110 | *** | -0.174 | *** | 0.246 | *** | 0.009 |  | 0.591 | *** | 0.591 | *** | -0.375 | *** | 0.735 | *** | — |  |
|  | | | | | | | | | | | | | | | | | | | | | | | | | | | | | | | | |  |
| *Note*. * *p* < .05. ** *p* < .01. *** *p* < .001 | | | | | | | | | | | | | | | | | | | | | | | | | | | | | | | | |  |

**The Results of Two Hierarchical Regression Analyses**

Please note that the results of the second steps of the two analyses are already reported in the article (see Table 5). Here the results of the first steps of the analyses are reported.

Analysis 1: The DT predicting global well-being

*F*(3, 1173) = 109.294, *p* < .001, *R²* = .218.

| Table S3  *Regression Coefficients* | | | | | | | | | | | | | | | | | |
| --- | --- | --- | --- | --- | --- | --- | --- | --- | --- | --- | --- | --- | --- | --- | --- | --- | --- |
|  | | | | | | | | | | | | | | **95% CI** | | | |
|  | |  | | **Unstandardized** | | **Standard Error** | | **Standardized** | | **t** | | **p** | | **Lower** | | **Upper** | |
|  |  | Machiavellianism |  | -1.008 |  | 0.211 |  | -0.146 |  | -4.772 |  | < .001 |  | -1.422 |  | -0.593 |  |
|  |  | Psychopathy |  | -1.591 |  | 0.218 |  | -0.226 |  | -7.308 |  | < .001 |  | -2.018 |  | -1.164 |  |
|  |  | Narcissism |  | 3.321 |  | 0.214 |  | 0.410 |  | 15.501 |  | < .001 |  | 2.901 |  | 3.741 |  |
|  | | | | | | | | | | | | | | | | | |

Analysis 2: Conceptions predicting global well-being

*F*(8, 1168) = 50.737, *p* < .001, *R²* = 0.258.

| Table S4  *Regression Coefficients* | | | | | | | | | | | | | | | | | | |  |
| --- | --- | --- | --- | --- | --- | --- | --- | --- | --- | --- | --- | --- | --- | --- | --- | --- | --- | --- | --- |
|  | | | | | | | | | | | | | | | **95% CI** | | | |  |
|  | |  | | **Unstandardized** | | **Standard Error** | | **Standardized** | | **t** | | **p** | | **Lower** | | **Upper** | |  |  |
|  |  | Eudaimonism |  | 0.005 |  | 0.019 |  | 0.006 |  | 0.250 |  | 0.802 |  | -0.033 | |  | 0.043 |  | |
|  |  | Inflexibility |  | 0.105 |  | 0.099 |  | 0.028 |  | 1.055 |  | 0.292 |  | -0.090 | |  | 0.299 |  | |
|  |  | Externality |  | -0.858 |  | 0.113 |  | -0.232 |  | -7.587 |  | < .001 |  | -1.080 | |  | -0.636 |  | |
|  |  | Fear |  | -0.704 |  | 0.112 |  | -0.192 |  | -6.287 |  | < .001 |  | -0.924 | |  | -0.484 |  | |
|  |  | Transformative |  | 0.580 |  | 0.100 |  | 0.168 |  | 5.806 |  | < .001 |  | 0.384 | |  | 0.776 |  | |
|  |  | Fragility |  | -0.730 |  | 0.101 |  | -0.196 |  | -7.240 |  | < .001 |  | -0.927 | |  | -0.532 |  | |
|  |  | Inclusive |  | 0.722 |  | 0.092 |  | 0.204 |  | 7.837 |  | < .001 |  | 0.541 | |  | 0.902 |  | |
|  |  | Valuing |  | -0.154 |  | 0.150 |  | -0.028 |  | -1.028 |  | 0.304 |  | -0.448 | |  | 0.140 |  | |
|  | | | | | | | | | | | | | | | | | | |  |
